# Prediction of the responsiveness to pharmacological chaperones: lysosomal human alpha-galactosidase, a case of study

**DOI:** 10.1186/1750-1172-5-36

**Published:** 2010-12-07

**Authors:** Giuseppina Andreotti, Mario R Guarracino, Marco Cammisa, Antonella Correra, Maria Vittoria Cubellis

**Affiliations:** 1 Istituto di Chimica Biomolecolare-CNR, Pozzuoli, Italy; 2 High Performance Computing and Networking Institute-CNR, Napoli, Italy; 3 Dipartimento di Biologia Strutturale e Funzionale, Universita' Federico II, Napoli, Italy; 4 Istituto di Biostrutture e Bioimmagini-CNR, Napoli, Italy

## Abstract

**Background:**

The pharmacological chaperones therapy is a promising approach to cure genetic diseases. It relies on substrate competitors used at sub-inhibitory concentration which can be administered orally, reach difficult tissues and have low cost. Clinical trials are currently carried out for Fabry disease, a lysosomal storage disorder caused by inherited genetic mutations of alpha-galactosidase. Regrettably, not all genotypes respond to these drugs.

**Results:**

We collected the experimental data available in literature on the enzymatic activity of ninety-six missense mutants of lysosomal alpha-galactosidase measured in the presence of pharmacological chaperones. We associated with each mutation seven features derived from the analysis of 3D-structure of the enzyme, two features associated with their thermo-dynamic stability and four features derived from sequence alone. Structural and thermodynamic analysis explains why some mutants of human lysosomal alpha-galactosidase cannot be rescued by pharmacological chaperones: approximately forty per cent of the non responsive cases examined can be correctly associated with a negative prognostic feature. They include mutations occurring in the active site pocket, mutations preventing disulphide bridge formation and severely destabilising mutations. Despite this finding, prediction of mutations responsive to pharmacological chaperones cannot be achieved with high accuracy relying on combinations of structure- and thermodynamic-derived features even with the aid of classical and state of the art statistical learning methods.

We developed a procedure to predict responsive mutations with an accuracy as high as 87%: the method scores the mutations by using a suitable position-specific substitution matrix. Our approach is of general applicability since it does not require the knowledge of 3D-structure but relies only on the sequence.

**Conclusions:**

Responsiveness to pharmacological chaperones depends on the structural/functional features of the disease-associated protein, whose complex interplay is best reflected on sequence conservation by evolutionary pressure. We propose a predictive method which can be applied to screen novel mutations of alpha galactosidase. The same approach can be extended on a genomic scale to find candidates for therapy with pharmacological chaperones among proteins with unknown tertiary structures.

## Background

Pharmacological chaperone (PC) therapy has been recently proposed as a promising strategy for the treatment of some genetic diseases. PC therapy exploits small molecules which can be administered orally, reach difficult tissues such as the brain and have low cost. The new approach relies on an unexpected finding: some molecules that at high dosage inhibit specific proteins, can, at low dosage, restore their activities in cells. They act as life jackets or chaperones for proteins that, although retaining the essential residues needed for activity, become unstable upon mutation and are degraded. These proteins are able to fulfill their duty if they are given the chance to survive long enough and get to the site where they are needed.

Pharmacological chaperone therapy, despite its novelty, has produced a few drugs which are already in clinical trials. The treatment of metabolic diseases with competitive inhibitors as chemical chaperons at sub-inhibitory intracellular concentrations was first proposed by Fan *et al *in 1999 [1]. They presented evidence that administration of Deoxy-galactonojirimycin (DGJ) at low concentration effectively enhanced mutant lysosomal alpha-galactosidase A [UNIPROT: AGAL_HUMAN] activities in lymphoblasts from Fabry patients with R301Q or Q279E mutations. Since then PC have been exploited for other lysosomal storage disorders such as Gaucher [2], Pompe [3], Tay-Sachs, Sandhoff [4], GM1 gangliosidosis [5,6], Niemann-Pick [7,8] and for the stabilization of a variety of non lysosomal proteins of medical interest such as the ATP binding cassette (ABC) family of transporters, G-protein-coupled receptors (GPCRs), tyrosinase, copper ATPase, p53 and carnitine transporters [9,10].

Fabry disease (FD) is X-linked and relatively frequent, 1-9 in 100000 [ORPHANET: orpha324, OMIM: 30150]. Different mutations of the gene encoding AGAL result in a wide phenotypic spectrum, with respect to age at onset, rate of disease progression, severity of clinical manifestations. Mutations with low or absent residual AGAL activity are generally observed in the classic infantile form of the disease. Patients with the late onset form of FD retain some AGAL activity and are asymptomatic until adult age when they develop cardiac and kidney problems. Nonetheless, as pointed out by Schaefer *et al *"clinical phenotype, age of onset and course of Fabry disease are very variable, even within the same family, which makes it difficult to define a genotype-phenotype relationship by analysing individual patients " [11]. Since the age of onset can be late and its complications, cardiac manifestations, stroke and chronic renal disease, are very similar to those of other very common disorders, FD could have been under diagnosed and an estimate as high as 1 in 3100 live births has been put forward [12].

FD offers an interesting case for studying the potentiality of PC because, since the pioneering work by Fan *et al *[1], responsiveness to PC has been assessed for a huge number of mutations, covering both early and late onset forms of Fabry disease [13-15]. A relatively large proportion of mutants, in particular among mutations associated with the late onset form of FD, recover activity when treated with DGJ. In this study, we correlated the responsiveness to DGJ with specific properties of the mutant AGAL sequences and developed a predictive protocol of general applicability to spot mutations which respond to PC.

## Results and Discussion

### Classification of mutants

To try to correlate the properties of AGAL mutants to their responsiveness to PC, we needed a set of mutations as large as possible. DGJ has been tested on a large proportion of AGAL mutants and we collected data from three different papers which reported the enzymatic activity for each mutation in the presence and absence of DGJ and the activity of the wild-type protein [13-15]. We gathered 96 different mutations in total which had been tested for responsiveness to DGJ. Responsiveness to DGJ is variable among mutations and we needed a precise threshold to define a binary label. We calculated the ratio between the activity of the mutants in the presence of the drug and the reference wild type activity measured in the absence of DGJ, for each study. This data (+DGJ/wild × 100) and the appropriate references are reported in additional files 1 and 2. We assigned responsiveness to two classes: 23 mutants were considered responsive because they recover at least 50% of normal activity in the presence of DGJ and 73 were considered non responsive. This conservative definition of responsiveness was adopted because the clinical indication of *GLA *mutations associated with FD is galactosidase activity less than 50% of the normal mean value in plasma [16].

### Structural characterization of mutants

Clinically important mutations can affect either the function or the structure of a protein: assessment of responsiveness to PC may depend critically on the correct classification of mutations. The first step in our analysis was the identification of the active site. For this purpose we detected pockets on the surface of AGAL, pdb code 3GXT, with the program CASTP [17,18]. Among these pockets we selected the one with the highest proportion of atoms belonging to conserved amino acids. We preferred this approach to direct identification of the residues in contact with galactose or DGJ in the crystal structures of the holo-enzyme [19-21].because galactose is a product of the enzyme obtained after the hydrolysis of a much larger substrate, globotriaosylceramide [22]. It might well be that residues in contact with galactose or with its analogue DGJ [19-21], represent only one part of the real active site.

**Figure 1:**
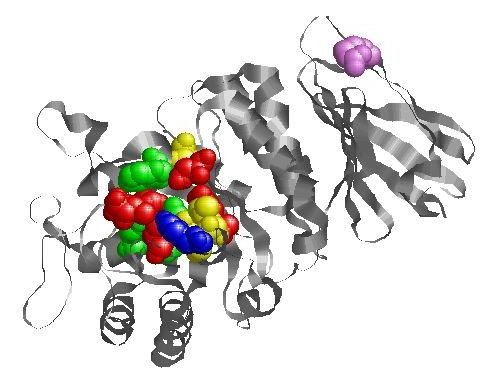
**The active site of human lysosomal alpha galactosidase**. A monomer of human lysosomal alpha galactosidase is shown as a ribbon. The pocket with the highest proportion of conserved amino acids includes four groups of amino acids: D92, D93, C142, D170, R227, D231 (in red), completely conserved and associated with mutations not responding to DGJ; Y207 (in blue), not conserved and associated with mutations not responding to DGJ; E203, L206, S297 (in yellow) conserved and not associated with mutations tested with DGJ; W47, Y134, K168 (in green) not conserved and not associated with mutations tested with DGJ. R363, the furthest site from active pocket where responsive mutations, R363C and R363 H, have been observed is shown in purple.

The pocket with the highest proportion of conserved amino acids includes four groups of amino acids: a) D92, D93, C142, D170, R227, D231 (red in Figure 1), residues completely conserved and associated with mutations not responding to DGJ; b) Y207 (blue in Figure 1), residue not conserved and associated with mutations not responding to DGJ; c) E203, L206, S297 (yellow in Figure 1) residues conserved and not associated with mutations tested with DGJ; d) W47, Y134, K168 (green in Figure 1), residues not conserved and not associated with mutations tested with DGJ. The pocket overlaps the residues which bind galactose or DGJ (D92, D93, K168, D170, E203, R227, D231) in the crystal structure of AGAL [21].

Mutations in the active site are inevitably non responsive: this is expected because DGJ acts only on the stability of the protein, and cannot restore the active site if this has been damaged. They represent 12% of the total number of non responsive mutations tested (Figure 1 and additional file 1).

The distance from the active site was also measured. Not only mutations that occur in the active site pocket, but also those close to it, tend to be non responsive (additional file 1) whereas it is possible that mutation occurring quite far from the DGJ binding site can be responsive to the drug as observed for example for R363H and R363C (R363 is coloured in purple in Figure 1). Indeed, distance from active site correlates with the percentage of recovered activity (+DGJ/wild × 100) with an r-value 0.16 and a p-value 0.02 (additional file 1).

AGAL has 5 intra-chain disulphide bonds and any tested mutation affecting a Cys involved in a bridge results in a non responsive protein. This result is also expected because disruption of a disulphide bond is usually highly destabilising and can prevent correct protein folding. It is worth noticing that not all Cys in AGAL are oxidised: Cys 90 and Cys 174 are not involved in a disulphide bridge, but they have not been found mutated in Fabry patients.

We then analysed other structure-derived independent features.

**Figure 2:**
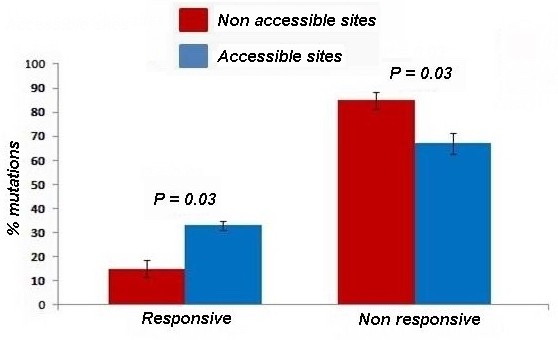
**Responsiveness to DGJ and solvent accessibility**. Occurrences of responsive or non responsive mutations in accessible (blue bars) or non accessible (red bars) residues are reported as percentage: we used a cut-off of 5.0% as a threshold of side-chain solvent accessibility. Differences between percentage shown in blue and red bars are statistically significant (p = 0.03).

Accessibilities to solvent of AGAL residues was measured both for the main chain atoms and the side chain atoms of each residue and are reported in additional file 1. Side chain accessibility correlates with recovered percentage activity (+DGJ/wild × 100) with an r-value 0.14 and a p-value 0.02 whereas main chain accessibility does not (additional file 1). We observe that mutations affecting exposed residues have higher chances of being rescued by PC (Figure 2), but, this holds only if we consider side chain accessibility. To assess the statistical significance of the differences observed in Figure 2 we performed the Wilcoxon rank sum test to reject the null hypothesis of equal medians at the default 5% significance level (P = 0.03).

We assigned each residue in AGAL to alpha helices, beta sheets or others (data in additional file 1). By others we mean any secondary structure different from alpha helices and beta sheets including coils, turns, helices 3-10, poly-proline, etc. Although some of these elements might well be associated with functional roles or with protein protein association [23,24], they are too rare in AGAL structure to be useful for predictive purposes. The percentages of responding residues in alpha helices, beta sheets and others reflect the percentage of responding mutations in the total data set suggesting that the occurrence in a specific secondary structure element does not determine the possibility of a mutation to be sensitive to PC. Although residues in beta sheet tend to respond when their side chains are exposed, paucity of data in each class prevents a reliable statistical analysis (data not shown).

We then used two programs, SDM [25,26] and MUPRO [27], to assign a stability score to the mutants, negative for unstable mutants and positive for stable ones, which is analogous to the free energy difference between a wild-type and mutant protein. The two programs provide independent assessment of protein stability because they rely on completely different approaches and do not reflect in any simple manner the structural features already analysed. SDM requires the knowledge of 3D-structure of AGAL and takes into account several structural features to predict the effect of mutations on protein stability. MUPRO does not require structural information on AGAL structure since it learns with a support vector machine method from the sequences deposited in ProTherm [28] database, a collection of numerical data of thermodynamic parameters for wild type and mutant proteins, and applies the derived rules to the sequence of an uncharacterised protein.

**Figure 3:**
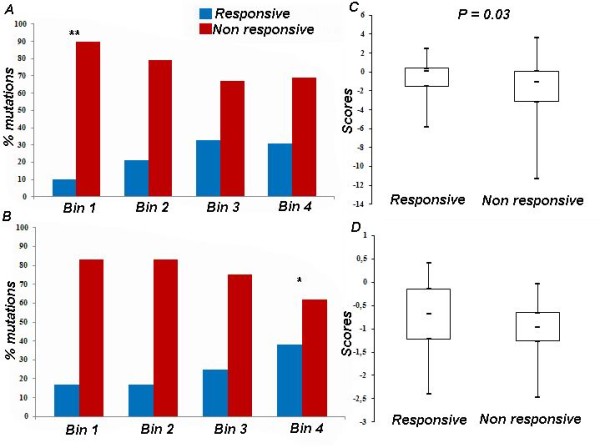
**Responsiveness to DGJ and mutant stability**. Mutants were divided into four equally populated bins (each including 25% mutations) of increasing predicted stability. For each bin a blue bar shows the percentage of responding mutations, a red bar the percentage of non responding ones. Panel A: bin 1 includes mutations with SDM scores ranging from -11.28 to -2.26; bin 2 from -2.26 to -0.86; bin 3 from -0.73 to 0.25; bin 4 from 0.25 to 3.65. Panel B: bin 1 includes mutations with MUPRO scores ranging from -2.46 to -1.25; bin 2 from -1.25 to -0.95; bin 3 from -0.95 to -0.60; bin 4 from -0.57 to 0.42. Bins with p = 0.01 or p = 0.04 are indicated with ** or * respectively. Box plots for the same data are shown in panel C for SDM and in panel D for MUPRO: the difference between the medians of SDM scores associated with responsive mutations and non responsive mutation is statistically significant (p = 0.03).

SDM scores correlate with the percentage of recovered activity (+DGJ/wild × 100) with an r-value 0.23 and a p-value 0.2 10^-3^(additional file 1) whereas MUPRO scores do not (additional files 1 and 2). Twenty per cent of the mutations obtain a score lower than -3 with SDM and all of these, but R363C, are non responsive. R363C is responsive since it reaches 57% of wild type enzyme activity upon treatment with DGJ, but gets a negative score, -5.84, by SDM. On the other hand, a different responsive mutation occurring at the same site, R363H, gets a positive score, 0.11(additional file 1). Therefore, SDM recognizes that a mutation of R363 can have a small effect on protein stability and hence can be potentially recoverable by PC, but over-estimates the damage caused by the specific substitution with a Cys. We ordered mutations by increasing SDM score and divided them into four equally populated bins: Figure 3 panel A shows that the percentage of responding mutations is low at low SDM scores and increases progressively as SDM score increases.

Similarly we sorted mutations by increasing MUPRO score and divided them into 4 bins: non responding mutations are more abundant in the bins associated with low MUPRO scores (Figure 3 panel B) but differences among bins are not as large as with SDM. The Pearson chi test confirms that the percentage of responsive mutations in the bin associated with low SDM scores (bin 1) is significantly different from expected (p = 0.01) whereas that in the bin associated with low MUPRO scores is not. On the other hand the percentage of responsive mutations in the bin associated with high MUPRO scores (bin 4) is significantly different from expected (p = 0.04) whereas that in the bin associated with high SDM scores is not.

We built box plots for the results obtained with the two programs: differences between respondent and non respondent mutations are more evident comparing the first quartile for SDM (Figure 3 panel C) or the third quartile for MUPRO (Figure 3 panel D). The Wilcoxon rank sum test rejects the null hypothesis of equal medians for SDM boxplots at the default 5% significance level (P = 0.03). Regarding MUPRO, we cannot assess the normal distribution of the two samples with sufficient confidence. However, we observed that the first quartile of SDM scores for non responding mutations occurs at -3.14, a value very close to the threshold of -3 below which, as already discussed, mutations are non responsive. On the other hand we observed that the third quartile of MUPRO scores for responding mutations occurs at -0.14 a value above which mutations are mainly responsive (additional file 2).

### Prediction of responsiveness

Analysis of AGAL structure reveals that three groups of mutations, those affecting the active site, those affecting disulphide bridges and those severely scored by SDM (< -3) are unlikely to be respondent to PC. However, a mutation which does not belong to any of these groups, can be either responsive or non responsive. In order to improve the usefulness of the model in patient therapy, we would like to predict responsiveness to DGJ for any AGAL mutation after having seen a number of training examples and this is, in statistical sense, a typical supervised classification task. Mutants have been labelled as respondent (23) or not respondent (73) and seven independent features derived from the analysis of 3D-structure of the enzyme and two features associated with their thermo-dynamic stability have been assigned to each of them as described before. Our goal is to find a mathematical function that given a set of features returns the correct class of the mutation. We tested all classification methods available in MATLAB-Arsenal developed by Rong Yan [29], which represent a large sample of all *de facto *standard classification algorithms. We compared the results obtained with MATLAB-Arsenal classifiers to those obtained with the ZeroR classifier. ZeroR classifier simply predicts the majority class in the training data. For example, if most of the training data are non respondent, ZeroR will predict all inputs as non respondent. Running ZeroR is necessary for determining a baseline performance as a benchmark for other learning schemes.

We found that although many structural derived features, distance from active site, side chain accessibility, SDM scores, disruption of disulphide bonds, correlate with percentage recovered activity (+DGJ/wild × 100 please see additional file 1), they are not useful to predict responsive mutations with an accuracy, precision or recall higher than the baseline ZeroR (data not shown). The classifier which performed significantly better than ZeroR is DecisionStump on MUPRO results, with 81% accuracy versus 24% of the baseline. Prediction of responsive mutations can be achieved with MUPRO because it assigns the highest scores preferentially to mutations which recover activity upon treatment with PC (additional file 2).

MUPRO predicts protein stability relying only on sequence information. This result encouraged us to rely on sequence alone and try and do better than MUPRO exploiting evolutionary conservation. In the first place we scored mutations using Blosum62, a matrix which considers only the pair of amino-acids involved regardless of the site where the mutation occurs. Performance of DecisionStump with Blosum62 scores (74% accuracy) is better than the baseline (24% accuracy), but much lower than that obtainable with MUPRO scores (81% accuracy). We decided to take into account the position where mutations occur in the protein using position specific substitution matrices (PSSM). PSSMs are built with PSI-BLAST [30], a program that uses as an input a set of homologous proteins, aligns the sequences and calculates amino acid substitution scores separately for each position in the multiple alignment. We carried out three independent experiments: we collected a first set of AGAL homologous proteins which includes very distant homologs with e-value as low as e-3, a second set which includes only close homologs with e-value higher than e-50 and a third set which includes only close orthologs, excluding the sequences derived by gene duplication. In fact, two paralogous lysosomal enzymes alpha-galactosidase A (AGAL_HUMAN; EC 3.2.1.22) and alpha-N-acetylgalactosaminidase (NAGAB_HUMAN; EC 3.2.1.49) exist in higher animals.

We run PSI_BLAST independently on the three sets and obtained different PSSM. The scores obtained with the PSSM built with close homologs of AGAL as well as those obtained with far homologs and close orthologs correlate with the percentage of recovered activity upon treatment with DGJ (+DGJ/wild × 100) respectively with r-value 0.44 and p-value <0.1 10^-3^; r-value 0.44 and p-value <0.1 10^-3^; r-value 0.32 and p-value 0.2 10^-3^. The scores obtained with PSSMs were used as inputs for DecisionStump to test their ability to predict responsive mutations. The PSSM built aligning close homologs (e-value <e-50) led to the best results: its performance (87% accuracy) is better than that obtained with the scores of MUPRO and, in particular, recall is much higher (49% versus 30%) which means that less false negatives are predicted (Table 1).

**Table 1:** Performance of prediction of DGJ responsiveness by DecisionStump on sequence derived features.

	accuracy	precision	recall
zeroR	0.240 ± 0.003	0.239 ± 0.003	1 ± 0

MUPRO	0.811 ± 0.001	0.555 ± 0.091	0.302 ± 0.017

Blosm62	0.737 ± 0.005	0.183 ± 0.057	0.176 ± 0.069

Close_homologs	0.867 ± 0.001	0.666 ± 0.125	0.489 ± 0.102

Far_homologs	0.786 ± 0.026	0.463 ± 0.033	0.364 ± 0.027

Close_orthologs	0.812 ± 0.001	0.477 ± 0.097	0.347 ± 0.062

PolyPhen	0.745 ± 0.028	0.179 ± 0.151	0.108 ± 0.058

Mutations were scored by the trivial classifier ZeroR to set a baseline, by MUPRO, by Bloum62 or by PSSMs derived by sets of sequences which include distant homologs (e-value > e-3), close homologs (e-value > e-50) or close orthologs (e-value > e-13).

The approach we used to predict responsiveness to PC is similar to that used by some programs to predict disease-associated mutations: results obtained with PolyPhen [31], which are reported in additional file 2 were used as inputs of DecisionStump classifier and allowed prediction of AGAL PC responsive mutations with 75% accuracy.

When developing a classifier, the numbers of responding mutations erroneously predicted as non responding (FN) as well as non responding mutations erroneously predicted as responding (FP) should be kept at a minimum. Therefore precision (TP/TP+FP) and recall (TP/TP+FN) are very useful to assess the performance of different methods. It is not surprising that precision and recall are lower than accuracy (TP+TN/TP+TN+FP+FN) in any case reported in Table 1 because correctly predicted non responding mutations (TN) increase accuracy but not precision and recall. Specific classes of non responding mutations are identified by structural analysis, and in general it is easier to predict TN than TP. DecisionStump based on the scores of the PSSM built with AGAL_HUMAN close homologs provides the highest precision and recall in comparison with the other tested methods (including PolyPhen). Nonetheless it should be emphasized that, regrettably, false positives and even more false negatives are predicted as implied by the fact that precision and recall values are well below 1 (Table 1).

Results obtained with sequence information alone do not improve if structure derived information are added as features for the classifiers in MATLAB-Arsenal [29](data not shown).

After this manuscript was submitted we became aware of three new AGAL mutations [32,33], C52Y, Y216C and G183A [32] which recover respectively <10%, 40% and 65% activity upon treatment with PC. This report offered us the opportunity for a test on mutations not included in the development of the method. C52Y disrupts a disulphide bonds and thus belongs to a class of mutations which are definitely unrecoverable. C52Y gets a negative score by SDM, -1.582, by MUPRO, -1.37 and by PSSM, -5, and is correctly predicted as non responsive. Y216C according to our conservative definition is non responsive since it does not reach 50% of wild type activity: it does not belong to the three groups of mutations which are definitely unrecoverable (i.e. it does not belong to the active site, it does not disrupt disulphide bonds, it is not severely destabilizing), but it gets a negative score -2.319 by SDM, -1.16 by MUPRO and -3 by PSSM and is correctly predicted as non responsive. G183A does not belong to the three groups of mutations which are definitely unrecoverable, it gets a positive score 2.33 by SDM and a negative score, -1.47, by MUPRO and is erroneously predicted as non responsive, because of a PSSM score -2. The PSSM score -2, is immediately below the threshold set by the classifier to minimize false negatives and false positives. Indeed, this score represents a twilight zone where the recovered activity (+DGJ/wild x100) of mutations can vary from 0% to 100% and for 19% of mutations is above 50%. We observed that with a few exceptions, responsive mutations erroneously predicted by the classifier (FN) either have a positive SDM score (as it is the case for G183A) or have a solvent exposed side chain.

## Conclusions

Mutations which occur at non exposed sites or in the active pocket are likely to be non responsive to DGJ as well as those compromising stability, but the complex interplay between functional and structural features on responsiveness makes difficult their exploitation for predictive purposes. However, the same features strongly tune evolutionary pressure at different sites in a protein. For this reason methods based on conservation alone can be exploited to predict responsiveness to PC: addition of structural features does not improve prediction because by measuring conservation we have already, implicitly, taken them into account. It is generally accepted that disease causing mutations are more frequent at conserved sites whereas non disease polymorphisms prevail at variable site and we demonstrated that this holds also for non responsive and responsive mutations. The score assigned to mutation by a PSSM takes into account the degree of conservation of the wild type amino acid in homologous sequences and the specific substitution that is introduced: if the new amino-acid is present in homologous species a less negative score is obtained. We have shown that high PSSM scores are associated with respondent mutations whereas low PSSM scores are associated with non respondent mutations and that this property can be exploited for predictions. It is not trivial, however the effect of the inclusion of homologs with a low percentage of identity in the construction of the PSSMs for at least three reasons:

1) sites that are critical for the function of enzymes in mammals or in metazoa might have varied in evolutionary distant species and many compensative mutations might have arisen

2) the inclusion of proteins in the twilight zone necessarily raise the possibility of introducing false homologs.

3) The inclusion paralogs can hinder functional important sites.

Our analysis proves that the choice of homologs for the construction of the PSSM is critical: paralogs can be included, but too distant homologs must be excluded.

Our method can be applied to predict PC responsiveness of novel and as yet untested mutations of alpha galactosidase. This is feasible and useful because a very high number of natural missense mutations have been found for AGAL, a middle sized protein of 429 aa: 146 are listed in Uniprot/Swissprot [34], 256 in the public version of Hgmd [35]. This figure might well rise if Fabry disease has been under diagnosed as it seems to be the case. In particular it can be predicted that new mutations associated with the late on-set form of the diseases, that are the most likely be respondent to DGJ will be found. In order to make a projection of the results obtainable with our method, we tested 299 mutations of AGAL listed in UniprotSwissprot and HGMD and we estimated that 40 mutations as yet untested with PC are likely to respond.

When a AGAL mutation not already tested *in vitro *for responsiveness to DGJ is encountered, clinicians can direct Fabry patients towards the therapy more likely to ameliorate their phenotype: a high PSSM score should suggest a beneficial effect of DGJ whereas a low PSSM score should suggest caution and direct towards enzymatic replacement [36,37]. DecisionStump classifier sets a threshold to minimize false negatives and false positives and allows, if required by clinicians, a clear cut indication for therapeutic intervention.

Although PC therapy will not substitute other therapies for FD such as enzymatic replacement [36,37], our results reinforce the idea that, at least in principle, these novel drugs, affordable and suitable for oral administration, have vast applicability.

Our approach to find candidates for PC therapy is not limited to alpha galactosidase, but can be extended on a genomic scale among proteins with unknown tertiary structures. Moreover due to the low cost associated with an in silico screening it meets regulators which look for ways to make it easier and cheaper for drug companies to develop treatments for rare diseases.

## Methods

### Construction of data set

A list of AGAL mutations was created and to each one a label, 1 for responsive and 0 for non responsive ones, was added. Original data of enzymatic activity in the presence or in the absence of DGJ were taken from three different papers [13-15]. The data is redundant since responsiveness of some mutants have been analysed by more than one group and we obtained in total 96 different tested mutations. In additional file 1 and 2 we calculated the percentage recovered activity, that is activity of the mutant in the presence of DGJ divided by the activity of wild type AGAL multiplied by 100 (+DGJ/wild × 100), and the appropriate reference.

The reference wild type activity, measured in the absence of DGJ was obtained for each mutation from the appropriate paper.

Mutants are responsive if in the presence of DGJ recover at least 50%, of normal activity and non responsive in the other case.

### Construction of PSSMs

We identified three sets of sequences: the first includes 163 homologs of AGAL with e-value < e-3 and identity ranging from 98% to 20% from GenBank CDS Translations, PDB, SwissProt, PIR, PRF; the second includes 13 homologs with e-value <e-50 and identity ranging from 95% to 30% from Uniprot/Swissprot; the third includes 15 orthologs with evalue e < e-150 and identity ranging from form 98%to 64% from translated GenBank, EMBL, DDBJ.

BLASTPGP (BLASTP2.2.18) in PSI-BLAST mode was used on each set flagging on the output of a checkpoint file (PSSM matrix) and selecting one of the following scoring matrices: Blosum80, Blosum62, Blosum45, Pam70 or Pam30.

Fifteen different PSSM were obtained and exploited in a second round of BLASTPGP to calculate net scores for AGAL mutants (mutant score PSSM(i)-Agal_wild score PSSM(i)) [30].

With any set of homologous proteins the best results (Table 1) were obtained selecting Blosum62 (BLASTPGP -d Blosum62); complete results for all PSSM are given in additional file 3.

The percentage of identical amino acids was also assessed aligning AGAL with 13 homologs with e-value <e-50 and identity ranging from 95% to 30% from Uniprot/Swissprot. At each position in the alignment we count the number of sequences sharing the same amino acid of AGAL and we divided by the total number of sequences in the alignment. Results in percentage are included in additional files 1 and 2.

### Active site identification

We detected active site residues with a procedure of general applicability inspired by the paper published by Dundas *et al *[17].

We detected cavities in AGAL structure, 3GXT, with the program CASTP [17,18] and we selected the pocket with the highest proportion of atoms belonging to conserved amino acids. To identify conserved residues we aligned 13 homologs with e-value <e-50 and identity ranging from 95% to 30% from Uniprot/Swissprot At each position in the alignment we count the number of sequences sharing the same amino acid of AGAL and we divided by the total number of sequences in the alignment.

All the residues lining that pocket, irrespective of their conservation, were considered to potentially belong to the active site.

### Prediction of mutants stability

MUPRO1.1 [27] was kindly provided by Dr J. Cheng: we run the program locally using as inputs only AGAL sequence, the position affected by the mutation, the original and the mutant amino-acid.

Models of all AGAL mutants are constructed using as template 3GXT [21] with the program ANDANTE which predicts side-chain conformations by use of environment-specific substitution probabilities and a high-quality rotamer library [38].

The asymmetric unit in 3GXT [21] contain the dimeric enzyme and monomers are not fully super-imposable as expected. For this reason each mutation was built in both chains.

SDM [25,26] uses a set of conformationally constrained environment-specific substitution tables and calculates the difference in the stability scores for the folded and unfolded state of each mutant and the wild-type protein. It uses as inputs the mutants generated with ANDANTE and the structure of the wild type enzyme.

The programs SDM and ANDANTE were kindly provided by T. Blundell and collaborators.

### Accessibility and secondary structure

Residue accessibility was calculated using PSA v2.0 (L.Cheng, unpublished) which uses the rolling probe algorithm [39]. For the threshold of solvent accessibility, we used a cut-off of 5.0% relative total side-chain or main chain accessibility.

The numbers of responsive or non responsive mutations with accessible side chain was divided by the total number of mutations with accessible side chain and multiplied by 100. In a similar way it was calculated the percentage of responding and non responding mutations with non accessible side chain.

The program PSA was kindly provided by T. Blundell and collaborators.

We assigned each residue in the wild type AGAL structure 3GTX to alpha helices, beta sheets or other with the program SEGNO [40].

### Supervised classification

We tested all classification methods available in MATLAB-Arsenal developed by Rong Yan [29]The accuracy of the methods has been assessed using tenfold cross validation. This means that we have divided all mutants for which the responsiveness is known in ten folds. At each step, one fold has been sorted out and used as a test. The remaining mutants have been used to train the classifier. In total, tests were repeated thirty times and values have been taken on average. Results are expressed as mean accuracy ((TP+TN)/TOT), which represents the mean percentage of correctly classified instances, precision (TP/(TP+FP)) and recall (TP/(TP+FN)).

When analysing structural features, mutations occurring on different chains had to be taken into account and therefore DecisionStump used 96 × 2 input data, when analysing sequence derived features, i.e. MUPRO, Blosum 62, PSSMs scores or PolyPhen PSIC score differences, DecisionStump used 96 input data.

### Statistical analysis

All statistical analyses were carried out with MATLAB.

To calculate the correlations and p values shown in additional files 1 and 2 we used corrcoef: this function returns a matrix R of correlation coefficients and a matrix of p-values for testing the hypothesis of no correlation. Each p-value is the probability of getting a correlation as large as the observed value by random chance, when the true correlation is zero.

To analyse the data shown in Figure 2 and 3 (panel C,D) we carried out the Wilcoxon test with the function ranksum. This function performs a two-sided rank sum test of the null hypothesis that data are independent samples from identical continuous distributions with equal medians, against the alternative that they do not have equal medians.

To evaluate the influence of solvent accessibility on PC responsiveness and to assess the statistical significance of the differences observed and differences expected by chance, we took a random sample of 30% in both exposed and non exposed population and counted the number of responding and non responding mutations. We repeated the randomization 100 times and calculated the differences between the percentage of responsive mutations in exposed and non exposed sites and the differences between the percentage of non responsive mutations in exposed and non exposed sites.

To test the hypothesis that distribution of Mupro and SDM scores among responsive and non responsive mutations is different from what could be expected by chance (Figure 3 panel A and B), we run the chi2test. We ordered mutations by increasing SDM (or MUPRO) score and divided them into four equally populated bins. The function used as inputs the percentages observed and expected in each bin and returned chi2 values and the associated p values.

## Abbreviations

DGJ: Deoxy-galactonojirimycin; FD: Fabry disease; FN: false negative; FP: false positive; PC: pharmacological chaperone; PSSM: position specific substitution matrix; TP: true positive; TN: true negative.

## Competing interests

The authors declare that they have no competing interests.

## Authors' contributions

GA and MVC designed the study and wrote the paper. MRG carried out preliminary statistical analysis and advised on MATLAB usage. MC and AC carried out structure based and sequence based analysis. All authors read and approved the final manuscript.

## Supplementary Material

Additional file 1 **Structure-based analysis of human lysosomal alpha galactosidase**. Original data of enzymatic activity in the presence or in the absence of DGJ were taken from three different papers: Benjamin *et al *2009, Shin *et al *2008, Shimotori *et al *2008 [13-15]. Percentage recovered activity, that is activity of the mutant in the presence of DGJ divided by the activity of wild type AGAL multiplied by 100 (+DGJ/wild × 100) is reported for each mutation with the appropriate reference. Responsiveness is set to 1 if (+DGJ/wild × 100) ≥50. The table contains SDM scores (SDM), % main chain accessibility (mc_access), % side chain accessibility (sc_access). Occurrence in alpha helix (alpha), beta strands (beta), other secondary structures (other), active site (as) or sites involved in disulphide bridges (ss) is indicated by 1. Distance from active site (as) is measured in angstrom, identity in close homologs (% ident) as percentage. The data were used as an input to calculate the correlation coefficients (R) and the p-values that are shown in a separate sheet (correlation and p-values). Each p-value is the probability of getting a correlation as large as the observed value by random chance, when the true correlation is zero. Click here for file

Additional file 2 **Sequence-based analysis of human lysosomal alpha-galactosidase**. Original data of enzymatic activity in the presence or in the absence of DGJ were taken from three different papers: Benjamin *et al *2009, Shin *et al *2008, Shimotori *et al *2008[13-15] Percentage recovered activity, that is activity of the mutant in the presence of DGJ divided by the activity of wild type AGAL multiplied by 100 (+DGJ/wild × 100) is reported for each mutation with the appropriate reference. Responsiveness is set to 1 if (+DGJ/wild × 100) ≥50. F_H, C_H and C_O indicate net scores derived from sets of sequences which include distant homologs (e-value > e-3), close homologs (e-value > e-50) or close orthologs (e-value > e-13) respectively. On each set of sequences PSI-BLAST was run selecting different scoring matrices, Blosum 45, Blosum62, Blosum80, Pam30, Pam70, therefore BL stands for Blosum, PM for Pam. Stability scores (MUPRO), Blosum62 scores (BL62), identity in close homologs (% ident), POLYPHEN predictions are also reported. The data were used as an input to calculate the correlation coefficients (R) and the p-values that are shown in a separate sheet (correlation and p-values). Each p-value is the probability of getting a correlation as large as the observed value by random chance, when the true correlation is zero. Click here for file

Additional file 3 **Performance of prediction of DGJ responsiveness by DecisionStump on sequence derived features**. Mutations were scored by PSSMs derived by sets of sequences which include distant homologs (e-value > e-3), close homologs (e-value > e-50) or close orthologs (e-value > e-13). On each set of sequences PSI-BLAST was run selecting different scoring matrices, Blosum 45, Blosum62, Blosum80, Pam30, Pam70. Click here for file

## References

[B1] Fan JQ Ishii S Asano N Suzuki Y Accelerated transport and maturation of lysosomal alpha-galactosidase A in Fabry lymphoblasts by an enzyme inhibitor Nat Med 1999 5 112 5 10.1038/4801 9883849

[B2] Khanna R Benjamin ER Pellegrino L Schilling A Rigat BA Soska R Nafar H Ranes BE Feng J Lun Y The pharmacological chaperone isofagomine increases the activity of the Gaucher disease L444P mutant form of beta-glucosidase Febs J 277 1618 38 10.1111/j.1742-4658.2010.07588.x 20148966 PMC2874831

[B3] Flanagan JJ Rossi B Tang K Wu X Mascioli K Donaudy F Tuzzi MR Fontana F Cubellis MV Porto C The pharmacological chaperone 1-deoxynojirimycin increases the activity and lysosomal trafficking of multiple mutant forms of acid alpha-glucosidase Hum Mutat 2009 30 1683 92 10.1002/humu.21121 19862843

[B4] Tropak MB Reid SP Guiral M Withers SG Mahuran D Pharmacological enhancement of beta-hexosaminidase activity in fibroblasts from adult Tay-Sachs and Sandhoff Patients J Biol Chem 2004 279 13478 87 10.1074/jbc.M308523200 14724290 PMC2904802

[B5] Caciotti A Donati MA d'Azzo A Salvioli R Guerrini R Zammarchi E Morrone A The potential action of galactose as a "chemical chaperone": increase of beta galactosidase activity in fibroblasts from an adult GM1-gangliosidosis patient Eur J Paediatr Neurol 2009 13 160 4 10.1016/j.ejpn.2008.03.004 18571950

[B6] Suzuki Y Chemical chaperone therapy for GM1-gangliosidosis Cell Mol Life Sci 2008 65 351 3 10.1007/s00018-008-7470-2 18202827 PMC11131625

[B7] Parenti G Treating lysosomal storage diseases with pharmacological chaperones: from concept to clinics EMBO Mol Med 2009 1 268 79 10.1002/emmm.200900036 20049730 PMC3378140

[B8] Beck M Therapy for lysosomal storage disorders IUBMB Life 62 33 40 20014233 10.1002/iub.284

[B9] Loo TW Clarke DM Chemical and pharmacological chaperones as new therapeutic agents Expert Rev Mol Med 2007 9 1 18 10.1017/S1462399407000361 17597553

[B10] Fan JQ A counterintuitive approach to treat enzyme deficiencies: use of enzyme inhibitors for restoring mutant enzyme activity Biol Chem 2008 389 1 11 10.1515/BC.2008.009 18095864

[B11] Schaefer E Mehta A Gal A Genotype and phenotype in Fabry disease: analysis of the Fabry Outcome Survey Acta Paediatr Suppl 2005 94 87 92 discussion 79 10.1080/08035320510031045 15895718

[B12] Spada M Pagliardini S Yasuda M Tukel T Thiagarajan G Sakuraba H Ponzone A Desnick RJ High incidence of later-onset fabry disease revealed by newborn screening Am J Hum Genet 2006 79 31 40 10.1086/504601 16773563 PMC1474133

[B13] Benjamin ER Flanagan JJ Schilling A Chang HH Agarwal L Katz E Wu X Pine C Wustman B Desnick RJ The pharmacological chaperone 1-deoxygalactonojirimycin increases alpha-galactosidase A levels in Fabry patient cell lines J Inherit Metab Dis 2009 32 424 40 10.1007/s10545-009-1077-0 19387866

[B14] Shin SH Kluepfel-Stahl S Cooney AM Kaneski CR Quirk JM Schiffmann R Brady RO Murray GJ Prediction of response of mutated alpha-galactosidase A to a pharmacological chaperone Pharmacogenet Genomics 2008 18 773 80 10.1097/FPC.0b013e32830500f4 18698230 PMC2657085

[B15] Shimotori M Maruyama H Nakamura G Suyama T Sakamoto F Itoh M Miyabayashi S Ohnishi T Sakai N Wataya-Kaneda M Novel mutations of the GLA gene in Japanese patients with Fabry disease and their functional characterization by active site specific chaperone Hum Mutat 2008 29 331 10.1002/humu.9520 18205205

[B16] Monserrat L Gimeno-Blanes JR Marin F Hermida-Prieto M Garcia-Honrubia A Perez I Fernandez X de Nicolas R de la Morena G Paya E Prevalence of fabry disease in a cohort of 508 unrelated patients with hypertrophic cardiomyopathy J Am Coll Cardiol 2007 50 2399 403 10.1016/j.jacc.2007.06.062 18154965

[B17] Dundas J Ouyang Z Tseng J Binkowski A Turpaz Y Liang J CASTp: computed atlas of surface topography of proteins with structural and topographical mapping of functionally annotated residues Nucleic Acids Res 2006 34 W116 8 10.1093/nar/gkl282 16844972 PMC1538779

[B18] CASTP http://sts.bioengr.uic.edu/castp/index.php

[B19] Garman SC Garboczi DN The molecular defect leading to Fabry disease: structure of human alpha-galactosidase J Mol Biol 2004 337 319 35 10.1016/j.jmb.2004.01.035 15003450

[B20] Guce AI Clark NE Salgado EN Ivanen DR Kulminskaya AA Brumer H Garman SC Catalytic mechanism of human alpha-galactosidase J Biol Chem 285 3625 32 10.1074/jbc.M109.060145 19940122 PMC2823503

[B21] Lieberman RL D'Aquino J A Ringe D Petsko GA Effects of pH and iminosugar pharmacological chaperones on lysosomal glycosidase structure and stability Biochemistry 2009 48 4816 27 10.1021/bi9002265 19374450 PMC2699628

[B22] Ishii S Yoshioka H Mannen K Kulkarni AB Fan JQ Transgenic mouse expressing human mutant alpha-galactosidase A in an endogenous enzyme deficient background: a biochemical animal model for studying active-site specific chaperone therapy for Fabry disease Biochim Biophys Acta 2004 1690 250 7 15511632 10.1016/j.bbadis.2004.07.001

[B23] Petock JM Torshin IY Weber IT Harrison RW Analysis of protein structures reveals regions of rare backbone conformation at functional sites Proteins 2003 53 872 9 10.1002/prot.10484 14635129

[B24] Cubellis MV Caillez F Blundell TL Lovell SC Properties of polyproline II, a secondary structure element implicated in protein-protein interactions Proteins 2005 58 880 92 10.1002/prot.20327 15657931

[B25] Worth CL Bickerton GR Schreyer A Forman JR Cheng TM Lee S Gong S Burke DF Blundell TL A structural bioinformatics approach to the analysis of nonsynonymous single nucleotide polymorphisms (nsSNPs) and their relation to disease J Bioinform Comput Biol 2007 5 1297 318 10.1142/S0219720007003120 18172930

[B26] Topham CM Srinivasan N Blundell TL Prediction of the stability of protein mutants based on structural environment-dependent amino acid substitution and propensity tables Protein Eng 1997 10 7 21 10.1093/protein/10.1.7 9051729

[B27] Cheng J Randall A Baldi P Prediction of protein stability changes for single-site mutations using support vector machines Proteins 2006 62 1125 32 10.1002/prot.20810 16372356

[B28] Gromiha MM An J Kono H Oobatake M Uedaira H Prabakaran P Sarai A ProTherm, version 2.0: thermodynamic database for proteins and mutants Nucleic Acids Res 2000 28 283 5 10.1093/nar/28.1.283 10592247 PMC102403

[B29] MATLAB-Arsenal http://www.informedia.cs.cmu.edu/yanrong/MATLABArsenal/MATLABArsenal.htm

[B30] Altschul SF Madden TL Schaffer AA Zhang J Zhang Z Miller W Lipman DJ Gapped BLAST and PSI-BLAST: a new generation of protein database search programs Nucleic Acids Res 1997 25 3389 402 10.1093/nar/25.17.3389 9254694 PMC146917

[B31] Ramensky V Bork P Sunyaev S Human non-synonymous SNPs: server and survey Nucleic Acids Res 2002 30 3894 900 10.1093/nar/gkf493 12202775 PMC137415

[B32] Filoni C Caciotti A Carraresi L Cavicchi C Parini R Antuzzi D Zampetti A Feriozzi S Poisetti P Garman SC Functional studies of new GLA gene mutations leading to conformational Fabry disease Biochim Biophys Acta 1802 247 52 19941952 10.1016/j.bbadis.2009.11.003 PMC3056268

[B33] Solis MA Pascual B Bosca M Ramos V Carda C Monteagudo C Torregrosa I Pons S Miguel A New mutation in female patient with renal variant of Fabry disease and HIV J Nephrol 23 231 3 20155722

[B34] Borglum AD Byskov A Cubellis MV Kruse TA An EcoRI polymorphism for the PLAUR gene Nucleic Acids Res 1991 19 6661 10.1093/nar/19.23.6661-a 1684424 PMC329266

[B35] HGMD http://www.hgmd.cf.ac.uk/ac/index.php

[B36] Mehta AB Anderson-Fabry disease: developments in diagnosis and treatment Int J Clin Pharmacol Ther 2009 47 Suppl 1 S66 74 20040315 10.5414/cpp47066

[B37] Hoffmann B Fabry disease: recent advances in pathology, diagnosis, treatment and monitoring Orphanet J Rare Dis 2009 4 21 10.1186/1750-1172-4-21 19818152 PMC2768700

[B38] Smith RE Lovell SC Burke DF Montalvao RW Blundell TL Andante: reducing side-chain rotamer search space during comparative modeling using environment-specific substitution probabilities Bioinformatics 2007 23 1099 105 10.1093/bioinformatics/btm073 17341496

[B39] Connolly ML Solvent-accessible surfaces of proteins and nucleic acids Science 1983 221 709 13 10.1126/science.6879170 6879170

[B40] Cubellis MV Cailliez F Lovell SC Secondary structure assignment that accurately reflects physical and evolutionary characteristics BMC Bioinformatics 2005 6 Suppl 4 S8 10.1186/1471-2105-6-S4-S8 16351757 PMC1866377

